# Physician clinical decision modification and bias assessment in a randomized controlled trial of AI assistance

**DOI:** 10.1038/s43856-025-00781-2

**Published:** 2025-03-04

**Authors:** Ethan Goh, Bryan Bunning, Elaine C. Khoong, Robert J. Gallo, Arnold Milstein, Damon Centola, Jonathan H. Chen

**Affiliations:** 1 Stanford Biomedical Informatics Research, Stanford University, Stanford, CA USA; 2 Stanford Clinical Excellence Research Center, Stanford University, Stanford, CA USA; 3 Division of General Internal Medicine (DGIM) at ZSFG, Department of Medicine, UCSF, San Francisco, CA USA; 4 Division of Clinical Informatics and Digital Transformation, UCSF, San Francisco, CA USA; 5 UCSF Action Research Center for Equity, UCSF, San Francisco, CA USA; 6 Center for Innovation to Implementation, VA Palo Alto Health Care System, Palo Alto, CA USA; 7 Communication, Sociology and Engineering, University of Pennsylvania, Pennsylvania, PA USA; 8 Division of Hospital Medicine, Stanford University, Stanford, CA USA

**Keywords:** Medical research, Diagnosis

## Abstract

**Background:**

Artificial intelligence assistance in clinical decision making shows promise, but concerns exist about potential exacerbation of demographic biases in healthcare. This study aims to evaluate how physician clinical decisions and biases are influenced by AI assistance in a chest pain triage scenario.

**Methods:**

A randomized, pre post-intervention study was conducted with 50 US-licensed physicians who reviewed standardized chest pain video vignettes featuring either a white male or Black female patient. Participants answered clinical questions about triage, risk assessment, and treatment before and after receiving GPT-4 generated recommendations. Clinical decision accuracy was evaluated against evidence-based guidelines.

**Results:**

Here we show that physicians are willing to modify their clinical decisions based on GPT-4 assistance, leading to improved accuracy scores from 47% to 65% in the white male patient group and 63% to 80% in the Black female patient group. The accuracy improvement occurs without introducing or exacerbating demographic biases, with both groups showing similar magnitudes of improvement (18%). A post-study survey indicates that 90% of physicians expect AI tools to play a significant role in future clinical decision making.

**Conclusions:**

Physician clinical decision making can be augmented by AI assistance while maintaining equitable care across patient demographics. These findings suggest a path forward for AI clinical decision support that improves medical care without amplifying healthcare disparities.

## Plain language summary

Doctors sometimes make different medical decisions for patients based on their race or gender, even when the symptoms are the same and the advice should be similar. New artificial intelligence (AI) tools such as GPT-4 are becoming available to assist doctors when making clinical decisions. Our study looked at whether using AI would impact performance and bias during doctor decision making. We investigated how doctors respond to AI suggestions when evaluating chest pain, a common but serious medical concern. We showed 50 doctors a video of either a white male or Black female patient describing chest pain symptoms, and asked them to make medical decisions. The doctors then received suggestions from an AI system and could change their decisions. We found that doctors were willing to consider the AI’s suggestions and made more accurate medical decisions after receiving this help. This improvement in decision-making happened equally for all patients, regardless of their race or gender, suggesting AI tools could help improve medical care without increasing bias.

## Abstract (web summary)

Goh et al. evaluate physician responses to GPT-4 assistance in standardized chest pain cases. The study demonstrates that physicians show willingness to modify their clinical decisions based on GPT-4 assistance, leading to improved clinical decision accuracy without introducing or exacerbating demographic biases in patient care.

## Introduction

The emergence of large language model (LLM) (e.g., GPT-4 and Med-PaLM2) generative AI systems challenge the very nature of medical practice and education^1^ when these automated systems demonstrate surprising accuracy on medical exam questions vs. human benchmarks^2^. Yet these systems still remain unfit for autonomous medical decision-making, given their propensity for confabulation, inconsistent behavior, lack of regulatory oversight, and the risk of unintended consequences such as exacerbating biases against underrepresented minority patients^3–5^.

Recent studies have revealed both the promise and complexity of integrating AI systems into clinical practice. In diagnostic reasoning tasks, a randomized trial of 50 physicians found that LLM assistance did not significantly enhance performance compared to conventional resources, despite the AI system alone demonstrating superior performance^6^. Conversely, in management reasoning tasks involving complex treatment decisions and risk assessment, LLM assistance significantly improved physician performance compared to conventional resources, though physician–AI collaboration still did not exceed the performance of the AI system alone^7^. While these studies demonstrate varying degrees of AI effectiveness across different clinical tasks, they leave open the question of whether physicians would be willing to change their initial clinical decisions after receiving AI advice, and how this might affect existing healthcare disparities observed in prior studies on individual vs. collective clinical decision making^8^.

In this study, we show that physicians are willing to modify their clinical decisions based on GPT-4 assistance, improving their accuracy scores from 47% to 65% in the white male patient group and 63% to 80% in the Black female patient group. This improvement occurs without introducing or exacerbating demographic biases, with both groups showing similar magnitudes of improvement (18%). 90% of physician participants surveyed indicate that AI tools will play a significant role in future clinical decision-making. These findings demonstrate that AI assistance can augment physician decision-making while maintaining equitable care across patient demographics.

## Methods

We employed a randomized pre-post intervention design to assess the impact of AI-assisted decision-making in healthcare. Fifty US-licensed attending physicians and resident physicians with training in a general medical specialty (internal medicine, family medicine, or emergency medicine) (Supplementary Table 1) were recruited through Stanford University email lists. Prior to participation, physicians were informed of the primary study aim: to evaluate how an AI recommender clinical decision support system might influence clinical decision-making. The consent process detailed that participants would review simulated clinical vignettes, propose management plans, and interact with a clinical decision support system. To maintain blinding, participants were not informed about the secondary aim of examining potential patient demographic influence on clinical decision-making. All participants consented to participate during this consent process.

**Fig. 1:**
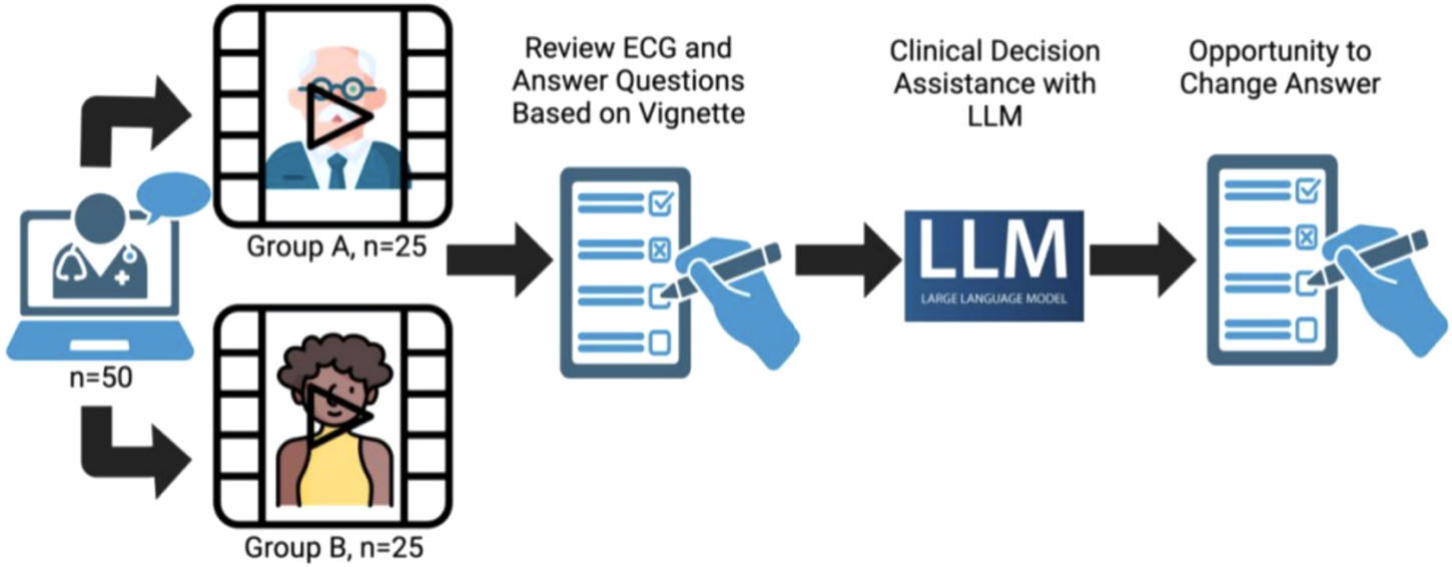
Study Design. Fifty US-licensed physicians were recruited for a remote video session where they were presented with a video of a standard patient actor depicting a case of chest pain in an outpatient setting. Participants were randomized to encounter an actor who was a white male or a Black female. The clinicians then responded to a series of four questions based on the vignette. For the first two questions, after providing their initial answers, they were presented with a pre-prepared LLM response based on the same vignette and questions. Clinicians were then offered an opportunity to modify their initial answers. For the final two questions, after their initial response, clinicians were allowed to directly interact with the LLM to ask any questions before considering whether or not to modify their answers.

Participants reviewed a video clinical vignette of a standardized patient complaining of chest pain (Fig. 1), with participants randomly assigned to have the case feature a white male or a Black female as used in a previous study^8^ demonstrating human biases in clinical interpretation. Participants were randomized using computerized block randomization (size 2). The randomization was single-blind, with only the study administrator (EG) knowing the video assignment. Participants were not aware of the randomization process or that different participants would see demographically varied presentations of the same clinical case. This design, approved by Stanford’s IRB, prevented priming effects from influencing baseline clinical decisions. The script the actors read stated:

“I’m so glad you were able to see me this afternoon. Ever since I retired a few years ago at 65, I’ve had time to try to get healthier. I know I’m overweight, so I’ve started to exercise more. After my walk this morning, I noticed a weird, tired feeling that made me feel a little short of breath. I sat down in my kitchen to get a sip of water and rest; it felt better a few minutes afterward. I also felt fine when I walked up the stairs to your office. The medical assistant who took my vital signs said everything looked great, and I’ve been taking the blood pressure and cholesterol medication every day. So, I don’t think it’s a big deal, but I want to make sure since my dad had a heart attack in his early 60’s.”

After reviewing the case vignette and associated ECG results developed for a previous study on clinical bias (Supplementary Note 2), participants answered four multiple choice clinical questions based on these vignettes (full case material and questions in Supplementary Notes 2 and 3), with the option of using any information resource available (e.g., MDCalc, Up-to-Date, PubMed) *except* for LLM AI systems. After each answer for questions #1 and #2 (based on the prior study’s vignette), participants reviewed a consistent, pre-generated ChatGPT+ (GPT-4) response using default system prompts from April 2023 (Supplementary Note 4) based on the case vignette information for questions #1 and #2. After each answer for questions #3 and #4, participants were allowed to directly interact with ChatGPT+ for assistance without any specific prompt guidance or pre-generated responses. Participants were given the option to change their answers after the above information interventions. The primary outcome measure was the accuracy of answers to the clinical decision questions, based on evidence-based literature review^8^. As a secondary measure, we studied the variance in accuracy before and after intervention between both groups.

### Statistical analysis

We analyzed results using R (v4.1.2) with a pre-specified linear mixed effects model (LMM) using the LME4 package (v1.1-34), with a random intercept for each participant. The model was first structured as: “Score (#correct out of 4 questions) ~ pre/post-recommendation + experimental-group + interaction-term + (1|participant)” with binary covariates. After modeling, the interaction term did not significantly improve the model (ANOVA, *p* = 0.88), and was dropped. Reported characteristics are from the LMM without an interaction term. The reference of the model covariates is pre-intervention and Group A (white male). Scores were treated as continuous variables. Model values were assessed at an unadjusted significance threshold of alpha = 0.05 using Satterthwaite’s *t*-test. Pre-study power calculations were done to estimate an adequate sample size and plan for adequate recruitment.

Following the completion of the clinical tasks, participants were asked to complete a survey to assess their perceptions of LLM tools like ChatGPT in healthcare (Supplementary Table 8). All participant interactions with ChatGPT+ (i.e., chat logs) were recorded and coded using an inductive qualitative data analysis approach to identify emergent themes^9,10^. This process was iterative, allowing categories to be refined for a precise representation of the interactions. E.G. independently coded the transcripts through readings of the transcripts. R.G. reviewed all transcripts subsequently to validate the coding.

### Reporting summary

Further information on research design is available in the Nature Portfolio Reporting Summary linked to this article.

## Results

Supplementary Table 5 reports a breakdown of participant scores for each individual question in both study groups, pre and post intervention. Notably, only 12% correctly answered the initial patient triage question in both study groups (“Full dose aspirin and refer to the emergency department”). This speaks to the need for clinical decision support systems to enhance even existing guideline-based recommendations. Among the other initial answers given by participants, 26% were under-triaged with an answer to “Start a daily baby aspirin and provide clear return precautions. Schedule the patient to return in one week.” 62% answered, “Start a daily baby aspirin and refer the patient for an urgent stress test within 2–3 days.” 0% over-triaged with an answer to “Provide a full-dose aspirin and contact cardiology for urgent cardiac catheterization.” Though not specifically evaluated in this study, some of the reasons that participants commented on for initially under-triaging included reassurance by the patient’s symptom resolution and the feeling of a reasonable follow-up plan in alternative answers. While some participants recognized the ECG T-wave changes, it appears that some missed the subtle changes in their initial assessment. With such low initial scores, question #1 is where many post-intervention accuracy gains were seen. In comparison, the accuracy scores for questions #3 and #4 (treatment choices with full interactive use of LLMs) were higher at baseline (48–84%) with consistent, but relatively less post-intervention improvement.

Table 1 reports the participants’ average scores on the clinical questions in each arm of randomization (white male patient vs. Black female patient) before and after exposure to GPT-4 responses. A statistical model showed significant differences in scores between the groups and pre- vs. post-LLM (Supplementary Tables 6 and 7).

**Table 1:** Clinician decision accuracy before and after LLM intervention

Average score metric	Group A (White Male) *N* = 25	Group B (Black Female) *N* = 25	The absolute difference between A and B
Initial (Out of 4)	1.9 (47%)	2.5 (63%)	0.62 (16%)
Post-LLM (Out of 4)	2.6 (65%)	3.2 (80%)	0.60 (15%)
Pre-Post Change (Out of 4)	+0.72 (18%)	+0.70 (18%)	−0.02 (−1%)

These results indicate that physicians are willing to change their clinical decisions based on responses from an automated large language model AI system, as opposed to anchoring on their initial decisions and skeptically refusing to be swayed by a computer-based response^11^. Moreover, doing so significantly improved the accuracy of their answers in this standardized cardiac chest pain scenario. This study primarily (re)tests the physician bias hypothesis in initial decision-making with a randomized-controlled design, while further testing and finding that physicians will change their answer judgments in response to AI-generated responses without introducing or exacerbating biases in this scenario.

A previous study^8^ established the validity of the clinical vignette and standardized patient videos, while also demonstrating bias in physician answers that could be mitigated through a crowdsourcing process. In contrast to the previous study, our statistical model, which adjusted for group and pre/post score, found that participants were more accurate when viewing the Black female video vs. the white male video (*p* < 0.01) (Supplementary Table 6). The reason for the differing results is unclear but could perhaps be attributed to the Hawthorne effect^12^, as participants completed this study in a virtual meeting setting while being observed by a researcher. It is also entirely possible that physician bias in medical decision-making is not a consistent phenomenon, having not been convincingly shown in other studies^13,14^. In either case, our statistical model (Supplementary Table 6) showed a significant improvement in participant scores post-intervention (*p* < 0.000001). This improvement was achieved without introducing or exacerbating any race or gender biases.

Different question types (triage, risk assessment, and treatment) were based on the previously established study and selected to mirror the variation in real-world clinical decisions that physicians encounter. Having a range of question types that involve judgment skills (risk and triage) vs. knowledge-based (evidence-based treatment selection) allowed us to assess the potential differential impact of potential bias and AI interaction methods on physician decision-making. Having a prepared LLM response for support in questions #1 and #2 ensured consistency in the user interaction, while the participant’s free open-ended use of ChatGPT+ for questions #3 and #4 allowed for additional qualitative analysis of the types of queries and interactions physicians would have with such a system in a live setting. The breakdown of the question accuracy results is summarized in Supplementary Table 5.

Table 2 describes categories of participant interactions with the AI chatbot when they were allowed freeform interaction with ChatGPT+ for treatment selection in questions #3 and #4, illustrating the multifaceted relevance of such technology in clinical decision-making settings. The usage patterns range from seeking clarification on guidelines and evidence-based practice to soliciting advice on specific patient scenarios. Specific examples of the participant’s chat log with the LLM are included, illustrating that many directly copy-pasted the clinical vignette or question content into the chat interface, while others asked further probing or summarized questions. While these findings are context-specific, they provide an initial understanding of the different types of physician/AI chatbot interactions and potential applications in clinical decision processes.

**Table 2:** Categorization of participant interactions with ChatGPT in a chest pain clinical vignette

Interaction	Description	Chat log transcript excerpt
1. Guideline and Evidence-based Practice Discussions	Physicians engaged with the AI to inquire about evidence supporting clinical decisions. This encompassed both specific clinical trials and current guidelines.	“What are the American Heart Association recommendations for a patient presenting with an NSTEMI and a TIMI score of 4 for initial medical therapy?” “What medical interventions have been shown to improve outcomes for NSTEMI?”
2. Patient Scenario-Based Inquiries	Physicians presented patient details to the AI, asking for advice or confirmation on management strategies.	“For a patient with chest pain, ST depressions, troponin elevations, what is optimal management?” “I have a 65-year-old patient with a history of hypertension and recent dyspnea on exertion who is now experiencing worsening chest pain, elevated troponin, and ST depressions on their EKG. There were no signs of heart failure or cardiogenic shock. In addition to potential catheterization, which of the following medical interventions are most likely to improve the patient’s clinical outcome?“
3. Validation of Own Knowledge or Beliefs	Physicians used AI to validate or challenge their own clinical knowledge or beliefs, especially by asking follow-up questions to clarify or expand on the information provided.	“I believe that nifedipine is contraindicated due to reflex sympathetic activation, is this correct?” “Why did you not include ARNIs?” “Why is sacubitril/valsartan not the right answer?”
4. Exploring New or Emerging Treatment Modalities	Physicians inquired about new or less familiar treatments and their benefits in specific clinical scenarios.	“What is the evidence supporting empagliflozin for post-MI?” “Does empagliflozin reduce mortality in patients with CAD after PCI?”
5. Comparative Analysis of Treatment Options	Physicians used AI to analyze and compare different treatment options.	“Can you list your reasoning for the correct answer: A. [list of medications] B. [list of medications]…?” “Are ACE inhibitors used in an acute NSTEMI?” “Are calcium channel blockers recommended in the acute management of patients with acute coronary syndrome?”

90% of participants indicated in a post-task survey (Supplementary Tables 8 and 9) that large language model tools like ChatGPT will play a significant role in healthcare for clinical decision making, with 66% rating the likelihood as ‘very likely’ and 24% as ‘somewhat likely’. Recommendations for improving the utility of AI chatbots in healthcare were varied but focused on increasing clinical relevance, such as by developing a healthcare-specific user interface and enhancing its ability to process and interpret patient information. Transparency in AI reasoning and decision-making processes was also a significant concern, with a call for AI chatbots to provide evidence-based citations for their recommendations.

A limitation of this study design is that the physician participants were given a video of a standardized patient case and an ECG image to review, whereas ChatGPT+ at the time of the study only allowed for text interaction, requiring it to be given a text-based summary of the clinical vignette. These text-based descriptions included “ECG showed T wave inversion/flattening” which made a subtle finding that many human participants might have missed on the ECG image into an explicit statement. It is conceivable, then, that the benefit of the LLM responses had more to do with its direct access to the ECG interpretation than the human participants did not. This is unlikely in this particular case study as the patient’s HEART cardiac risk score that drove the risk triage questions would add up to the same “moderate” category regardless of whether or not the 1 point for ECG changes was identified.

Additional challenges in the context of using LLMs are that they exhibit variation in their outputs based on different prompts, algorithmic updates, and underlying randomness. To maximize internal validity within this study, we developed the vignette content with varying case prompts (including with vs. without demographic information) and used default system prompts while repeatedly querying the LLM to assess for variation in outputs. While the specific wording of the outputs always differed with each request, repeated prompting during the study period up to August 2023 confirmed that the produced LLM outputs remained similar in meaning and suggested answers (and not showing different suggestions depending on the patient’s stated race or gender that has been observed under other specific adversarial scenarios)^15^. This is important to track as repeating the same example case prompts on versions of the ChatGPT system as of May 2024 does NOT always produce the same answer recommendations in the generated responses (sometimes recommending option B for question #1 to schedule an urgent stress test within 2-3 days rather than option C to refer the patient to the emergency department).

This study should not be used to conclude the general capability of LLMs or humans in medical decision-making or bias in general. The present study was limited to a single standardized clinical vignette to isolate the human-computer interaction phenomenon and is not intended to represent the broad scope of medical practice. We chose this case vignette for internal validity and consistency with a previous study^8^ that established the case vignette as a basis to assess for biases through video-recorded standardized patients and evidence-based reference answer evaluations. Further evaluations can be done across a broader set of cases or even broad collections of live patient scenarios^16^. This study remains critical to move beyond the many studies evaluating LLM (vs. human) performance on medical questions to directly administer, observe, and evaluate the interaction and impact of augmenting human physicians with emerging LLM generative AI systems.

The results of this study indicate that physicians are receptive to AI chatbot-based suggestions in their clinical judgment across a spectrum of clinical question types (e.g., risk assessment, triage, treatment). Within a controlled standardized vignette around cardiac chest pain assessment, a large language model AI system was able to significantly alter physician medical decisions, improving accuracy without introducing or exacerbating existing demographic bias.

## Supplementary information

Supplementary Information

Description of Additional Supplementary Files

Supplementary Data 1

Reporting Summary

## Data Availability

LLMs outputs are included in the supplement with the prompts used. Transcript chat logs, raw score table, and individual survey responses are available upon request.
