# Supplementary material for: Physician clinical decision modification and bias assessment in a randomized controlled trial of AI assistance
Source: Commun Med (Lond). 2025 Mar 4;5:59. doi: 10.1038/s43856-025-00781-2 (PMC11880198; doi:10.1038/s43856-025-00781-2)
Supplement: Supplementary file 2 — Description of Additional Supplementary Files [file 43856_2025_781_MOESM2_ESM.pdf]

Description of Additional Supplementary Files

File name: Supplementary Data 1

File description: This file contains anonymized data and responses from all study participants.
